# *N*‐ to *C*‐Glycoside Rearrangement of Uridine 5′‐Phosphate in Two Enzymatic Steps for the Production of Pseudouridine 5′‐Phosphate

**DOI:** 10.1002/bit.29037

**Published:** 2025-05-28

**Authors:** Martin Pfeiffer, Franziska Guld, Bernd Nidetzky

**Affiliations:** ^1^ Institute of Biotechnology and Biochemical Engineering Graz University of Technology Graz Austria; ^2^ Austrian Centre of Industrial Biotechnology (ACIB) Graz Austria

**Keywords:** cascade biocatalysis, pseudouridine, pseudouridine 5’‐phosphate, purine/pyrimidine nucleotide 5’‐phosphate nucleosidase (EC 3.2.2.10), ribose 5’‐phosphate, uridine 5’‐phosphate

## Abstract

Pseudouridine (**Ψ**) is an essential building block of synthetic RNA for medical applications, so methods for its efficient production receive increased interest. Reverse reaction of the **Ψ**‐5′‐phosphate (**ΨMP**) *C*‐glycosidase, that is, d‐ribose 5‐phosphate (**Rib5P**) + uracil (Ura) → **ΨMP** + H_2_O, allows for the installment of the core β‐*C*‐riboside structure of **Ψ** in a completely selective and efficiently equilibrium‐driven single‐step transformation. However, providing the **Rib5P** substrate is challenging for process development and optimum solutions can vary depending on the specific production tasks considered. Here, we exploited the less known activity of purine/pyrimidine nucleotide 5′‐phosphate nucleosidase (PpnN; EC 3.2.2.10) to cleave uridine 5′‐phosphate (**UMP**), a relatively expedient starting material for **ΨMP** synthesis, under release of **Rib5P** and **Ura**. Using linear cascade transformation in two enzymatic steps performed in one pot, we demonstrate rearrangement of **UMP** into **ΨMP** (yield: ≥ 95%) and thereby obtain the *C*‐riboside product at the solubility limit (∼1.5 mol/L) in a productivity of 2.9 × 10^2 ^g/L/h. We show that a previously reported R341A‐Y347A double variant of *Escherichia coli* PpnN (RY) exhibited ∼5‐fold higher specific activity toward UMP, and was ∼12‐fold less sensitive to **Rib5P** inhibition, than the wild‐type enzyme. Under conditions of *C*‐glycosidase applied in twofold excess over PpnN to minimize the effect of **Rib5P** inhibition, cascade reaction with RY compared to wild‐type PpnN still gave ∼5‐fold enhanced productivity. In summary, we present a new synthetic route to **ΨMP** via *N*‐ to *C*‐glycoside rearrangement of **UMP**. Compared to earlier approaches of cascade biocatalysis for **ΨMP** production from uridine or **UMP**, this new route is streamlined due to the direct release of **Rib5P** from the **UMP** substrate catalyzed by PpnN.

## Graphical abstract

The authors present a streamlined cascade reaction for one‐pot N‐C rearrangement of uridine‐5′‐phosphate (**UMP**) to pseudouridine‐5′‐phosphate (**ΨMP**; a key mRNA vaccine building block), in two enzymatic steps catalyzed by **UMP** nucleosidase (EC 3.2.2.10) and **ΨMP** *C*‐glycosidase (EC 4.2.1.70). **ΨMP** was obtained in > 95% yield and with a productivity of 2.9 × 10^2 ^g/L/h. **ΨMP** production (∼ 1.5 g) at ∼ 93% purity involved low E‐factor of just 3.0.

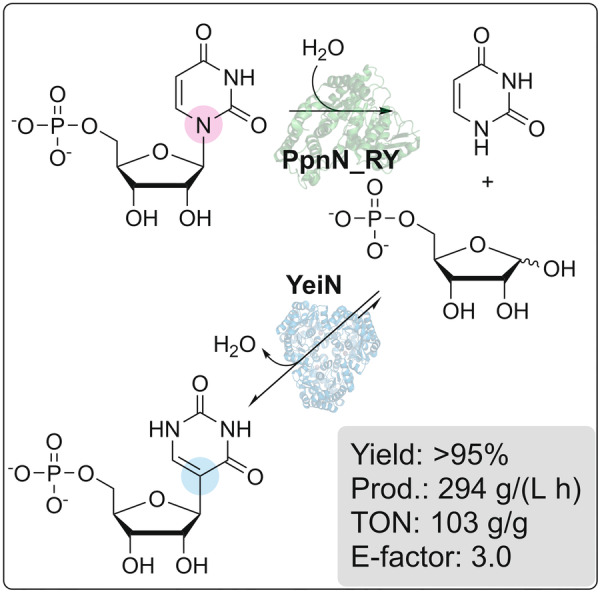

**Abbreviations**
m1Ψ: N^1^‐methyl‐**Ψ**
ppGpp: guanosine 3′,5′‐bispyrophosphate
PpnN: purine/pyrimidine nucleotide 5′‐phosphate nucleosidase (EC 3.2.2.10)
Rib: d‐ribose
Rib5P: d‐ribose 5‐phosphate
RY: R341A‐Y347A double variant of PpnN
UMP: uridine 5′‐phosphate
Ura: uracil
YeiN: **ΨMP**‐*C*‐glycosidase (EC 4.2.1.70)
Ψ: pseudouridine
ΨMP: pseudouridine 5′‐phosphate
ΨTP: **Ψ** 5′‐triphosphate

## 1 Introduction

**Figure 1:**
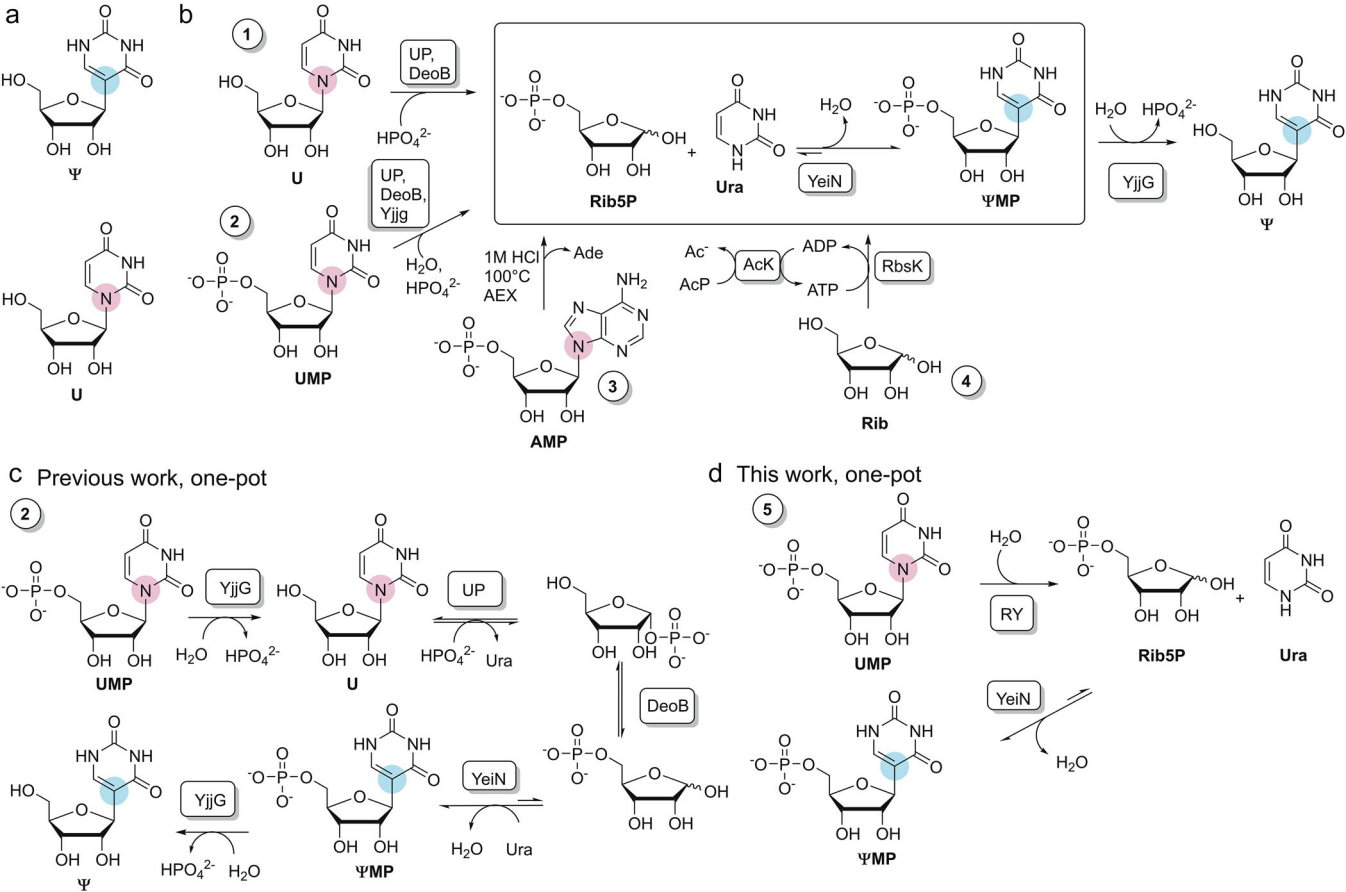
Pseudouridine (**Ψ**), the *C*‐nucleoside isomer of uridine (**U**), and its synthesis by biocatalytic cascade transformations of different substrates. Panel a: chemical structure of **Ψ**. Panel b: Synthetic routes from **U** (1, Pfeiffer et al. 2023), **UMP** (2, Pfeiffer et al. 2023), **AMP** (3, Riley et al. 2021) and **Rib**/**Ura** (4, Pfeiffer and Nidetzky 2020; Ribar et al. 2024). The assigned number is later used as route identifier in Table 1. The abbreviations/names used are: RY, R341A‐Y347A double variant of PpnN (EC 3.2.2.10); UP, uridine phosphorylase (EC 2.4.2.3); DeoB, phosphopentomutase (EC 5.4.2.7); YjjG, nucleoside 5′‐phosphate phosphatase (EC 3.1.3.5); RbsK, ribokinase (EC 2.7.1.15); AcK, acetate kinase (EC 2.7.2.1); YeiN, **ΨMP**‐ *C*‐glycosidase (EC 4.2.1.70); Ade, adenine; ATP, adenosine 5′‐triphosphate; ADP, adenosine 5′‐diphosphate; AcP, acetyl phosphate; Ac, acetate; AEX, intermediate work‐up by anion exchange chromatography.

Pseudouridine (**Ψ**, Figure 1A) is the natural 5‐β‐*C*‐riboside isomer of uridine (Spenkuch et al. 2014). **Ψ** is found in RNA where certain **U** positions are isomerized post‐transcriptionally (Borchardt et al. 2020; Cerneckis et al. 2022; Hamma and Ferré‐D'Amaré 2006). **Ψ** and its N^1^‐methylated derivative (**m1Ψ**) have important uses in synthetic mRNA for medical applications (Karikó et al. 2008). As an example, the current mRNA vaccines against COVID‐19 involve uniform substitution of **U** by **m1Ψ** (Andries et al. 2015; Nance and Meier 2021). The growing importance of mRNA‐based therapeutics brings with it the requirement of robust (bio)process technologies for mRNA production. The supply chain of **m1Ψ** and its immediate synthetic precursor **Ψ** is critical to the manufacture of mRNA by in vitro transcription (Light and Lexchin 2021). Current synthetic routes to **Ψ** involve multi‐step procedures requiring protecting groups and lack the required stereoselectivity for β‐*C*‐riboside formation exclusively (Hanessian and Machaalani 2003; Van Rijssel et al. 2015). Therefore, new methods for a more efficient and more sustainable production of **Ψ** attract increased attention. Cascade biocatalysis developed around C‐C condensation by **Ψ**‐5′‐phosphate (**ΨMP**) *C*‐glycosidase as the core reaction is promising to generate important process innovation. The *C*‐glycosidase is a microbial enzyme whose biological role is the hydrolysis of **ΨMP** to recycle d‐ribose 5‐phosphate (**Rib5P**) and uracil (**Ura**) into the metabolism (Preumont et al. 2008). The C‐C coupling between **Rib5P** and **Ura** involves large driving force from thermodynamics, so the *C*‐riboside formation, which is formally the reversion of hydrolysis, proceeds in almost quantitative yield, even in water (Pfeiffer and Nidetzky 2020). Hydrolysis of **ΨMP** is functional in vivo due to continued removal of **Rib5P** by central metabolism (Preumont et al. 2008). Used under optimized conditions in vitro, however, the reverse *C*‐glycosidase reaction gives **ΨMP** in superior concentration (≥ 1 mol/L) and productivity (≥ 93 mmol/L/h) (Pfeiffer et al. 2023; Ribar et al. 2024). Process concepts for biocatalytic synthesis differ in how **Rib5P** is supplied to the *C*‐glycosidase reaction (Figure 1B). They also differ in whether **Ψ** or **ΨMP** is the actual target product. **Rib5P** was obtained by phosphorylation of d‐ribose performed in one pot together with **ΨMP** synthesis. Dephosphorylation of **ΨMP** in a separate enzymatic step was used to prepare **Ψ** (Figure 1B). **Rib5P** was obtained additionally from adenosine 5′‐phosphate by chemical hydrolysis performed in a separate step with product isolation (Riley et al. 2021). Lastly, **Rib5P** was released together with **Ura** from **U** or **UMP** using a one‐pot four‐enzyme cascade transformation to produce **Ψ**, as shown in Figure 1B. Synthesis of **Ψ** was demonstrated at gram scale via cascade reaction from **U** (Pfeiffer et al. 2023).

**Ψ** and **ΨMP** are useful products in their own right. Synthetic routes to **m1Ψ** have been developed from **Ψ** (Bhattacharya et al. 1995; Earl and Townsend 1977; Reichman et al. 1977). mRNA production by in vitro transcription requires the 5′‐triphosphate‐activated derivatives of **Ψ** or **m1Ψ** (Nance and Meier 2021). In this context, **ΨMP** can be considered as already primed for further enzymatic phosphorylation to yield **ΨTP** (Pfeiffer et al. 2023; Pfeiffer and Nidetzky 2020).

In the current study, we identified **UMP** as an interesting (i.e., relatively inexpensive) starting material for the production of **ΨMP**. Our earlier work has shown the use of **U** and **UMP** for synthesis of the unphosphorylated product **Ψ**. It is important to note that **ΨMP** was not accessible via the one‐pot cascade reaction used previously (Figure 1C). Here, we realized the opportunity for streamlining the enzymatic cascade reaction through direct release of **Rib5P** from **UMP** (Figure 1D). The enzyme for this reaction (purine/pyrimidine nucleotide 5′‐phosphate nucleosidase, PpnN; EC 3.2.2.10) is known in principle (Sévin et al. 2017), but has not been explored for applied biocatalysis. We show the PpnN from *Escherichia coli* for an efficient cleavage of **UMP** into **Rib5P** and **Ura**. PpnN characterization performed in this study identified inhibition by **Rib5P** as an important criterion in selecting the suitable enzyme for the cascade reaction with the *C*‐glycosidase. We showed that a previously reported double variant of PpnN (R341A‐Y347A, in short: RY; Zhang et al. 2019) exhibits ∼5‐fold higher specific activity toward **UMP** and shows ∼12‐fold lower inhibition by **Rib5P** than the wild‐type enzyme. Combining the PpnN double variant and the *C*‐glycosidase in a one‐pot transformation in two steps, we show a clean and highly efficient rearrangement of **UMP** into **ΨMP** (yield: ≥ 95%). The *C*‐riboside product was obtained at its solubility limit (∼1.5 mol/L) in a productivity of 2.9 × 10^2 ^g/L/h. Overall, therefore, integration with the PpnN reaction significantly expands the synthetic scope of the *C*‐glycosidase reaction for providing the critical **Ψ**(**MP**) precursors to mRNA building block production.

## 2 Methods

### 2.1 Materials

**UMP** (disodium salt) and other chemicals were from Carl Roth (Karlsruhe, Germany). The enzymes used were from *E. coli*: **ΨMP** *C*‐glycosidase (YeiN; UniProt accession number: P0ADR8) and PpnN (UniProt accession number: P33025).

### 2.2 Enzyme Preparation

YeiN was prepared by reported procedures using expression in *E. coli* NiCo21(DE3) and purification by metal affinity chromatography (Pfeiffer et al. 2023). The enzyme was equipped with an *N*‐terminal His_6_‐tag.

Standard cloning techniques were used to obtain plasmid expression vectors for PpnN and RY. Synthetic gene of wild‐type PpnN was obtained commercially. Mutations resulting in the substitution of Arg341 by Ala and Tyr347 by Ala to generate RY were introduced by site‐directed mutagenesis. The procedures used are given in full in the Supporting Information. For expression, the genes were cloned into pET28a(+) vector to obtain enzyme equipped with *C*‐terminal His_6_‐tag. The vectors were transformed into NiCo21(DE3) competent *E. coli* and selected on LB agar plates with 0.05 mg/mL kanamycin. Enzymes were prepared by standard expression and purification by metal affinity chromatography. Details of the methods used are given in the Supporting Information.

Purity of the enzymes used was confirmed by PAGE (Figure S1).

### 2.3 Enzyme Characterization

#### 2.3.1 General Procedure of Initial‐Rate Analysis

Unless otherwise mentioned, all reactions were performed in 50 mM HEPES buffer (pH 7.5) at 40°C in a total volume of 100 µL. An Eppendorf Thermomixer (Hamburg, Germany) was used and agitation was set at 300 rpm. Reactions were started with enzyme solution (≤ 2% of total volume) added to the temperature‐equilibrated substrate solution. The substrate concentrations were varied and are specified in the individual experiments. Samples of 15 µL were taken at certain times, quenched with 65 µL of mixture of methanol and water (1:1, by volume), centrifuged for 5 min at 27,150 × *g*, and analyzed by HPLC. Initial rates were determined from the linear range of product formation (or substrate consumption) under conditions of substrate conversion below 20%. In activity assays, the unit (U) refers to an initial rate of 1 µmol/min of product released or substrate consumed.

#### 2.3.2 YeiN Assay

Initial rates of **ΨMP** release were determined at 15 mM of each **Ura** and **Rib5P** using 0.025 mg/mL enzyme. The YeiN preparation showed a specific activity of 7.0 U/mg, consistent with earlier literature on the enzyme (Pfeiffer and Nidetzky 2020).

#### 2.3.3 PpnN Assay

Initial rates of **Ura** release were determined using 30 mM **UMP** with 0.4 mg/mL wild‐type enzyme or 0.1 mg/mL RY. The specific activities of the enzymes are reported under Results. The standard assay was performed at 40°C and pH 7.5. Temperature profiles (30°C–60°C, pH 7.5) and pH profiles (40°C, pH 6.0–8.0) of the activity were also recorded.

#### 2.3.4 Kinetic Characterization of PpnN

Initial rates of **Ura** release were determined at 6 varied concentrations of **UMP** (40°C, pH 7.5). The **UMP** concentration range was chosen individually for wild‐type PpnN and RY. Inhibition by **Rib5P** was studied at three varied concentrations of inhibitor, measuring initial rates in dependence of the **UMP** concentration. The standard Michaelis–Menten equation with or without term for competitive inhibition (Equation 1) by **Rib5P** was used to fit the data by nonlinear least‐squares regression (SigmaPlot V10.0; Erkrath, Germany). *V* (mM/min) is the initial rate, *V*_max_ (mM/min) is the maximum initial rate, *K*_m_ (mM) is the Michaelis constant, and *K*_i_ (mM) is the inhibition constant.

(1) $$V=V_{max}\left[UMP\right]/(K_{m}\left(1+\left[Rib5P\right]/K_{i}\right)+\left[UMP\right]).$$

The relationship *V*_max_ = *k*_cat_ [E] was used to determine the turnover frequency (*k*_cat_). [E] is the molar concentration of enzyme subunit which was determined from the protein concentration and the molecular mass (wild‐type PpnN: 51,794 Da; RY: 51,440 Da).

#### 2.3.5 Hydrolysis of UMP

Reactions were performed as described above using 100 mM **UMP** as the substrate. The consumption of **UMP** and release of **Ura** in dependence of the incubation time were measured by HPLC. Wild‐type PpnN and RY were used. The resulting progress curves were fitted on the basis of Equation 1 and the mass balance of the reaction (**UMP** → **Ura** + **Rib5P**), using the differential equation solver of Berkeley Madonna 10.2.8. At certain times, sample was taken from the reaction and diluted into the assay for PpnN activity to determine whether enzyme inactivation had occurred.

### 2.4 Cascade Reaction for Synthesis of ΨMP

Reactions were performed in 50 mM HEPES buffer (pH 7.5) containing 15 mM MnCl_2_ at 40°C. Unless otherwise mentioned a total volume of 300 µL was used. Incubations were made in a Thermomixer with agitation at 300 rpm. **UMP** was used in a concentration from 100 to 1700 mM. The concentrations of RY and YeiN were adjusted to substrate concentration in the range 0.5–4.0 mg/mL. The mass ratio of RY and YeiN was also varied. The conditions are reported under the Results with the respective experiment. Samples taken over time were analyzed by HPLC. **UMP**, **Ura**, and **ΨMP** were quantified. It was verified that reported results are consistent on the basis of close mass balance.

### 2.5 Preparative Synthesis of ΨMP

The **ΨMP** synthesis was performed in a 10 mL round‐bottom flask that was placed in a water bath for temperature control at 40°C. Magnetic stirring (IKA RCT basic, Staufen, Germany) at 900 rpm was used for mixing. **UMP** (disodium salt; 1.84 g, 5 mmol; final concentration: 1.0 M) and MnCl_2_ (0.009 g; final concentration: 15 mM) were dissolved in 3.0 mL doubly distilled water. The pH was adjusted to 7.4 with 1 M NaOH. The final volume was set to 5.0 mL by adding water. The **UMP** concentration was checked by absorbance at 260 nm. The reaction was started with enzyme: 1.0 mg/mL RY, 2.0 mg/mL YeiN.

After completion of conversion of **UMP** (HPLC, 97%), the mixture was heated (100°C for 5 min), precipitate was centrifuged off (5 min at 27,150 × *g*) and the supernatant was filtered using Amicon Ultra‐15 Centrifugal Filter Units (Millipore, Billerica, MA, USA). The permeate was frozen in liquid nitrogen and lyophilized with an Alpha 1‐4 LSCplus freeze dryer (Martin Christ Gefriertrocknungsanlagen GmbH, Osterode am Harz, Germany) connected to a VACUUBRAND (Wertheim, Germany) vacuum pump model RZ 6. The resulting product (1.6 g) was **ΨMP** in purity of 93% (by mass).

**Yield**: 1.60 g (4.35 mmol), 87%, colorless solid, C_9_H_11_Na_2_N_2_O_9_P [368.15].

^**1**^ **H NMR** (400 MHz, D_2_O) δ = 7.69 (s, 1H, H‐3), 4.60 (d, *J* = 3.4 Hz, H‐5), 4.07 (t, *J* = 5.9 Hz, 1H, H‐7), 4.02 (d, *J* = 4.0 Hz, 1H, H‐6), 3.93 – 3.84 (m, 2H, H‐8, H‐9), 3.73 (dd, *J* = 11.6, 5.0 Hz, 1H, H‐9). Glycerol (10 mol %).

^**13**^ **C NMR** (101 MHz, D_2_O) δ = 165.20 (C_q_, C‐1), 152.77 (C_q_, C‐2), 141.02 (CH, C‐3), 111.54 (C_q_, C‐4), 81.31 (d, *J* = 8.3 Hz CH, C‐8), 78.60 (CH, C‐5), 74.29 (CH, C‐6), 69.77 (CH, C‐7), 63.00 (d, *J* = 4.4 Hz, CH_2_, C‐9).

### 2.6 Analytics

#### 2.6.1 NMR

Spectra were recorded on a JEOL (Freising, Germany) JNM‐ECZL 400 MHz spectrometer (^1^H: 399.78 MHz, ^13^C: 100.53 MHz, ^31^P: 161.83 MHz) and were processed with JEOL software. Details are given in the Supporting Information.

#### 2.6.2 HPLC

Reversed‐phase ion‐pairing method with UV detection at 260 nm was used (Supporting Information). **UMP**, **Ura**, and **ΨMP** were baseline separated and quantified individually using authentic standards as reference.

#### 2.6.3 Concentration Determination

Absorbance was measured with a DeNovix (Wilmington, DE, USA) DS‐11 Spectrophotometer and concentrations were calculated with the molar extinction coefficient. **Ura** (258 nm, 8.3 mM^−1^cm^−1^), **UMP** (262 nm, 10 mM^−1^cm^−1^), **ΨMP** (262 nm, 7.5 mM^−1^cm^−1^), YeiN (280 nm, 10,095 M^−1^cm^−1^; 32,909 Da), and PpnN/RY (280 nm 10,095 M^−1^ cm^−1^; 32,909 Da).

## 3 Results and Discussion

### 3.1 Characterization of PpnN

Wild‐type PpnN and RY variant were obtained as purified protein preparations (Figure S1) from *E. coli* overexpression cultures in yields of ∼125 mg/L. Both enzymes retained their full activity for over 1 month when stored frozen at −20°C. RY was chosen because its activity for hydrolysis of xanthine 5′‐phosphate was previously reported to be ∼2.3‐fold higher than that of the wild‐type enzyme (Zhang et al. 2019). RY differs from the wild‐type enzyme furthermore in that its activity is no longer controlled allosterically by the metabolic messenger guanosine 3′,5′‐bispyrophosphate (ppGpp) and instead of a tetramer it is present as a monomer in solution (Zhang et al. 2019). The specific activity of RY measured with **UMP** (30 mM) at 40°C and pH 7.5 was 9.8 (±1.6; *N* = 6) U/mg which exceeds that of wild‐type PpnN under the same conditions by ~5‐fold (2.6 ± 0.8 U/mg; *N* = 6). Temperature and pH were set to match the conditions used previously for the *C*‐glycosidase cascade reactions (Pfeiffer and Nidetzky 2020; Pfeiffer et al. 2023). However, temperature and pH profiles of **UMP** hydrolysis (Figures S2 and S3) show that the conditions used fell squarely into the suitable operational range of both wild‐type enzyme and RY. The pH‐profile of the wild‐type enzyme at 40°C was extended slightly more into the alkaline range (pH 8.0) than the pH profile of RY at the same temperature (Figure S2). The optimum pH range for both enzymes was 6.0–7.0. However, pH 7.5 was still useable with ~75—80% of the maximum activity retained in both wild‐type PpnN and RY (Figure S2). For both enzymes, the temperature profile at pH 7.5 showed maximum at 50°C (Figure S3). PpnN reaction performed at pH 7.5°C and 40°C was thus supported. Slight differences in the pH‐ and temperature‐dependencies of activity for the two enzymes may arise from the fact that the wild‐type PpnN is a functional tetramer whereas RY is a monomer (Zhang et al. 2019).

Initial rates of **UMP** hydrolysis showed hyperbolic dependence on the substrate concentration for both enzymes (Figure S4). Kinetic parameters revealed that RY (*k*_cat_ = 13.4. ± 1.4 s^−^^1^) was ∼5‐fold faster than the wild‐type PpnN (*k*_cat_ = 2.6 ± 0.2 s^−1^). However, apparent binding of **UMP** was ∼15‐fold weaker for RY (*K*_m_ = 15.5 ± 6.6 mM) compared to wild‐type PpnN (*K*_m_ = 1.1 ± 0.4 mM). In terms of their catalytic efficiency (*k*_cat_/*K*_m_), the two enzymes were therefore similar (wild‐type: 2.3; RY: 0.9).

### 3.2 Hydrolysis of UMP

**Figure 2:**
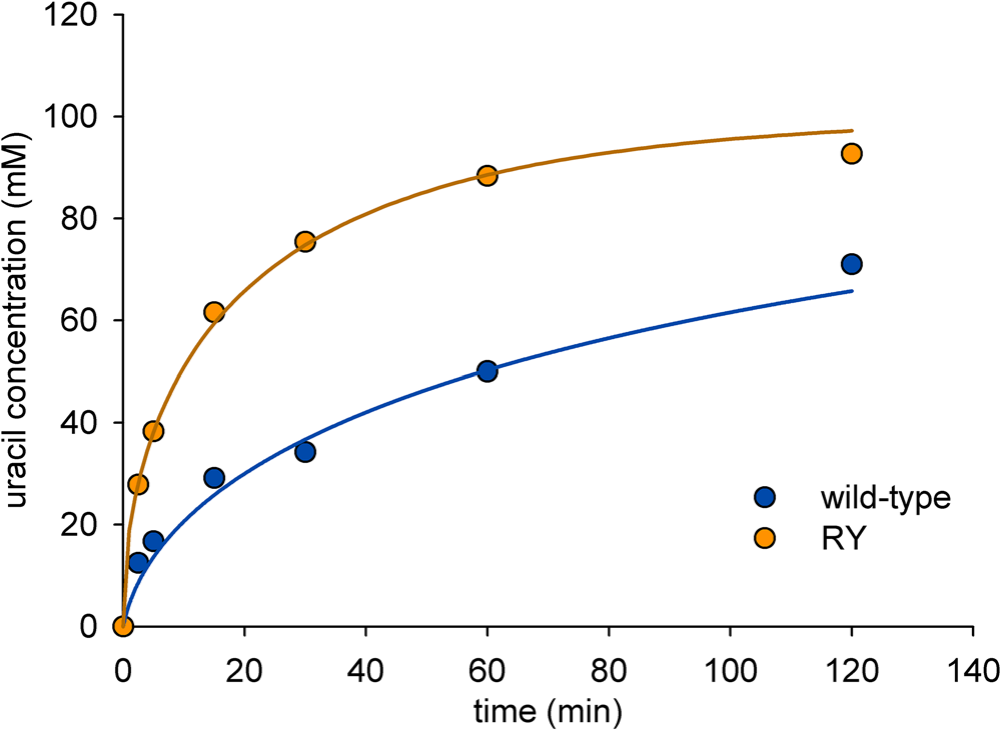
Hydrolysis of **UMP** by wild‐type PpnN and RY. Each enzyme was used at 2.0 mg/mL. Symbols show the data and lines are fits with Equation 1 using the *K*_i_ for **Rib5P** as the adjustable parameter.

Wild‐type PpnN and RY were compared in time‐course experiments of **UMP** hydrolysis (Figure 2). When applied at the same protein concentration (2.0 mg/mL), RY gave faster conversion than wild‐type PpnN. Moreover, the rate of the wild‐type reaction decreased faster with progressing substrate conversion than that of RY (Figure 2). The RY reaction achieved 92% hydrolysis of the **UMP** used (100 mM) in only ∼120 min. The corresponding wild‐type reaction leveled off after the same time, so that it would have required a disproportional extension of the reaction time (≥ 400 min) to reach the same conversion. We determined that both enzymes retained their activity fully for 3 h under the conditions used, thus ruling out that difference in enzyme stability was a relevant factor. We speculated that inhibition by **Rib5P** might be important and pursued the idea with two types of experiment.

First, we applied a Michaelis–Menten kinetic model with competitive product inhibition by **Rib5P** to fit the time courses in Figure 2. The *K*_m_ and *k*_cat_ were set constant using the values from the initial‐rate characterization, and the inhibition constant (*K*_i_) for **Rib5P** was fitted. The *K*_i_ was estimated as 1.2 mM for RY which was 12‐fold higher than the estimated *K*_i_ of 0.1 mM for the wild‐type enzyme. The *K*_m_/*K*_i_ ratio exceeded a value of unity for both enzymes (wild‐type: 11; RY: 13), suggesting rather strong inhibition by the accumulating product. In general, one considers a *K*_m_/*K*_i_ ratio of 0.1 or smaller to be favorable for enzymatic production largely unaffected by product inhibition. However, the inhibitory effect in the hydrolysis of **UMP** was clearly more severe in wild‐type PpnN than RY (Figure 2). We accounted for **Rib5P** inhibition of PpnN in the design of the subsequent cascade reactions for **ΨMP** synthesis from **UMP**. The *C*‐glycosidase YeiN was always used in twofold excess over PpnN with the idea to remove **Rib5P** faster than it was released from **UMP**.

**Figure 3:**
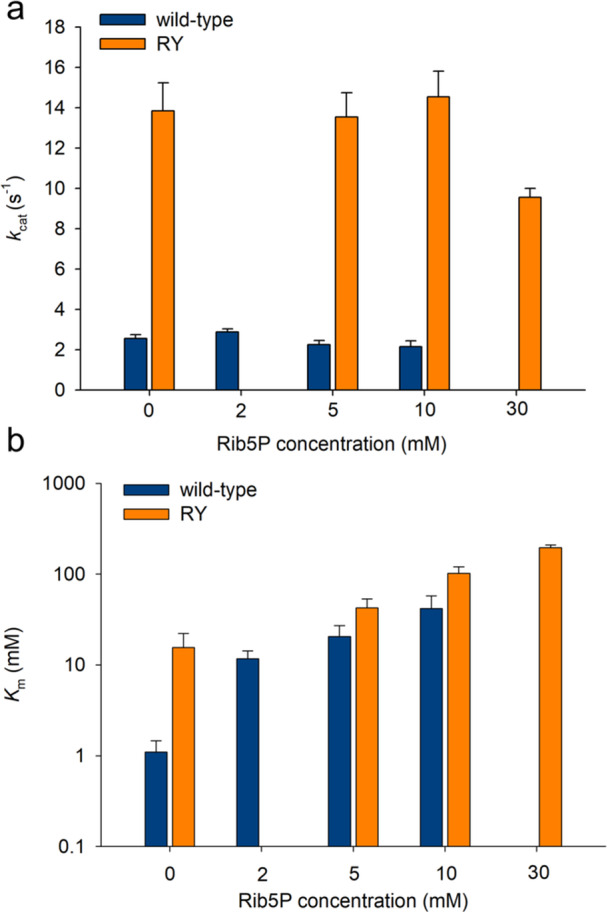
Kinetic parameters of wild‐type PpnN and RY obtained from inhibition studies with **Rib5P**. Panel a shows the dependence of *k*_cat_ on Rib5P concentration. Panel b shows the dependence of *K*_m_ on Rib5P concentration. The underlying experimental data and their associated fits are shown in Figure S5.

In a second approach, we studied **Rib5P** inhibition directly (Figures S5 and S6). Results show that with both wild‐type PpnN and RY, the inhibition by **Rib5P** vanished at saturating concentration of **UMP** so that the estimated *k*_cat_ was not affected substantially (Figure 3a). By contrast, the estimated *K*_m_ increased strongly for both enzymes (Figure 3b), suggesting that the inhibition was competitive in each case. The result is significant considering that the wild‐type PpnN structure exhibits an allosteric site for the binding of the secondary messenger ppGpp. The allosteric site is located at the subunit‐subunit interface of the tetrameric wild‐type enzyme (Zhang et al. 2019). It is disrupted in the RY variant that is shown from earlier work to be unable to oligomerize into a tetramer (Zhang et al. 2019). Inhibition by **Rib5P** in wild‐type PpnN might therefore also arise due to primitive affinity of the ppGpp site for binding phosphorylated sugars including **Rib5P**. This possibility is ruled out by the data shown. The effect of **Rib5P** on *K*_m_ differed between wild‐type enzyme and RY. At 10 mM **Rib5P**, the wild‐type *K*_m_ was increased 40‐fold (1.1 to 41 mM) whereas the RY *K*_m_ was increased only 6.7‐fold (15 to 100 mM). Nonlinear fit of the kinetic data (Figures S5 and S6) gave a *K*_i_ estimate of 1.2 ± 0.1 mM for RY. The corresponding *K*_i_ estimate for the wild‐type enzyme was 0.1 mM. Both values are consistent with the *K*_i_ estimates obtained from time course fits of **UMP** hydrolysis (Figure 2).

### 3.3 One‐Pot Rearrangement of UMP Into ΨMP

**Figure 4:**
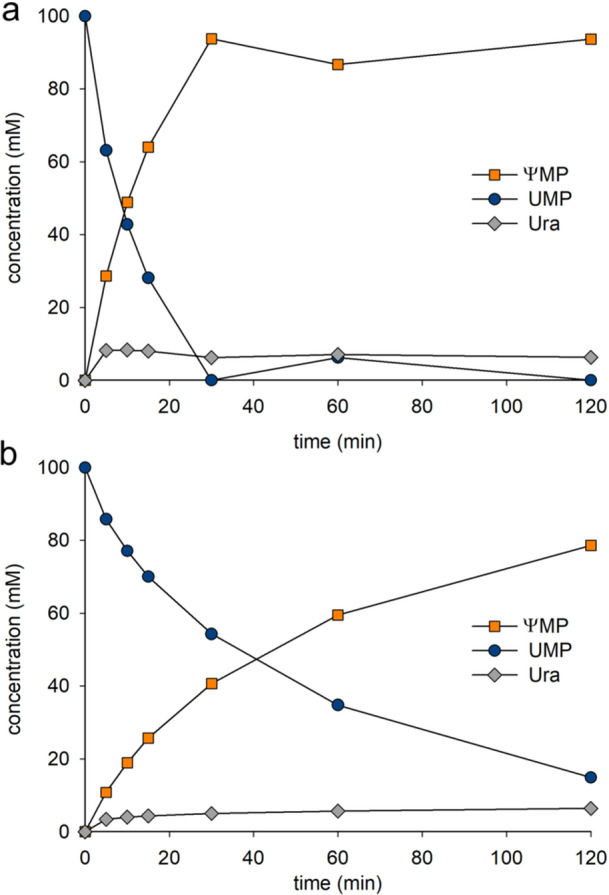
PpnN enzyme comparison for synthesis of **ΨMP** in the cascade reaction with YeiN. Panel a shows the reaction course of RY (0.50 mg/mL), panel b shows that of wild‐type PpnN (0.50 mg/mL). YeiN was used at 1.0 mg/mL in both reactions. Reactions were performed in 50 mM HEPES buffer (pH 7.5) containing 15 mM MnCl_2_ at 40°C. Symbols show the data. Lines are used to visualize the trend.

**Figure 5:**
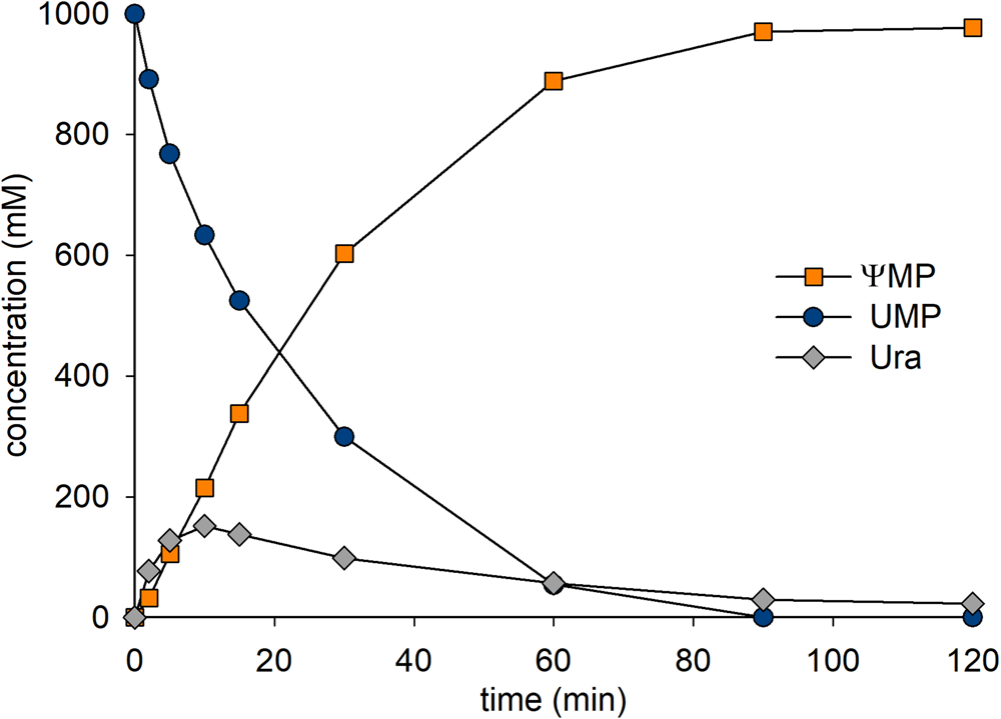
Preparative synthesis of **ΨMP** in a scaled‐up reaction at 5.0 mL. Conditions: 1.00 M **UMP**, 1.0 mg/mL RY, 2.0 mg/mL YeiN, reaction in water (15 mM MnCl_2_) with pH adjusted to 7.5.

The time course of **ΨMP** release from **UMP** (100 mM) by cascade reaction of RY and YeiN is shown in Figure 4a. Substrate was used completely in 30 min and the corresponding (≥ 95%) amount of product was released. **Ura** was present in low amount (~5%) in accordance with close mass balance for the overall transformation. The linear cascade reaction (Figure 1d) achieved a net rearrangement of **UMP** into **ΨMP** in high yield. The corresponding time course of the cascade reaction of wild‐type PpnN and YeiN is shown in Figure 5. Although the overall rearrangement was equally selective (≥ 95% yield of **ΨMP** on **UMP** converted) as it was in the RY reaction, the substrate conversion progressed more slowly (≥ 2.5‐fold) when the wild‐type PpnN was used. Comparing the two reactions at 80% conversion of **UMP**, the productivity of the RY reaction was ∼5‐fold that of the reaction of wild‐type PpnN. The results affirm the benefit of reaction optimization that results from the use of RY instead of wild‐type PpnN.

The RY reaction proceeded to completion (**UMP** conversion ≥ 95%) with only small decrease in the reaction rate with progressing depletion of the substrate (Figure 4a). This indicates that the effect of **Rib5P** inhibition was low, certainly lower than it appears to have been in the reaction with wild‐type PpnN which involved a curved time course that approached full conversion only slowly (Figure 4b). The importance of kinetic pull by the YeiN reaction to remove the **Rib5P** released from **UMP** was demonstrated in an experiment in which the mass ratio of RY and YeiN was varied at constant total protein loading. Results in Figure S7 support the idea of using RY as the limiting enzyme. When YeiN was present in a mass amount same as, or twofold higher than, the amount of RY the reaction proceeded at the full speed possible (Figure S7). Lowering the YeiN amount below the used amount of RY resulted in a substantial decrease in the conversion, likely because of increased inhibition of RY by the accumulating **Rib5P**.

### 3.4 Reaction Intensification for ΨMP Production

We used the cascade reaction by RY and YeiN to assess **ΨMP** production at elevated concentration of the **UMP** substrate (500–1700 mM). Keeping the mass ratio of RY and YeiN constant at 1:2, the enzyme concentrations were adjusted to allow for a substrate conversion of ∼80% or greater in 2 h. The solubility of **UMP** was found to be limited at ∼1.5 M under the conditions used. Figure S8 shows the reaction time courses obtained. The excellent yield and selectivity of the overall rearrangement were retained at high **UMP** concentration. To avoid spontaneous precipitation of **UMP** or **ΨMP**, we decided to limit the starting concentration of **UMP** to 1 M. Scale up of the reaction from 100 µL to 5.0 mL allowed for preparative synthesis of **ΨMP**. As shown in Figure 5, the reaction proceeded to 95% yield and involved superior productivity of 900 mM/h or in mass units, 294 g/L/h. Following removal of the enzymes by ultrafiltration, the product solution was lyophilized to obtain 1.6 g of **ΨMP** in a purity of 95% (HPLC) and an isolated yield of 85%. The chemical structure of the isolated **ΨMP** was verified by NMR (Figures S9,S10).

Table 1 summarizes key metrics of reaction efficiency (Kaspar et al. 2021; Meissner and Woodley 2022) for **ΨMP** production from **UMP** by the RY‐YeiN cascade transformation and it uses these metrics to compare different approaches of cascade biocatalysis (Figure 1b,c; Routes 1, 2, and 4) for the synthesis of **ΨMP** or **Ψ**. The synthetic scope of the RY‐YeiN transformation (Figure 1d) is unique because **ΨMP** is obtained from **UMP**. Previously a one‐pot cascade reaction of four enzymes was used to convert **UMP** but the product was **Ψ** (Figure 1c). Routes to **ΨMP** start from **U** or **Rib**. In comparison to these other routes, the RY‐YeiN reaction is streamlined to involve fewer steps and enzymes (2 instead of 3 or 4). The total enzyme usage is lowered up to threefold and the productivity is increased up to 16‐fold. The conversion yield (≥ 95%) and the final product concentration (950 mM) are both excellent. The process operation is simple without the requirement for pH control that was needed in the **ΨMP** synthesis from **Rib**. The facile work‐up resulted in a low E‐factor of 3.0 (Supporting Information, E‐factor calculation file) and the product purity was high (≥ 93%, by mass). **Ura** and **Rib5P** were present at below 3% (by mass). Further present were in mass% MnCl_2_ (0.5%) and glycerol (0.5%) as a contamination form the centrifugal filtration. The mass‐based TON of the total enzymes used in the reaction reached 100 g product/g enzyme. Note that enzyme recycling was not considered at this stage to further enhance the TON. Based on multiple criterions of synthetic efficiency as shown in Table 1 (Supporting Information, E‐factor calculation file), the **ΨMP** production from **UMP** by coupled RY and YeiN was on par, or even outperformed, the earlier reported cascade transformations to deliver **ΨMP** or **Ψ**. In terms of the substrate costs, **UMP** is an expedient starting material sold in kg quantities; its anticipated market price for an upscaled production is similar to that of **U** or **Rib** and **Ura**. Assuming perfect scalability from the mL to the L scale, the reaction volume required to obtain 1 kg **ΨMP** is calculated as 3.1 L.

**Table 1:** Route comparison for the biocatalytic production of ΨMP and Ψ.

Route identifier^a^	Enzymes^b^	Substrate	Product	Productivity (g/L h)/(mM/h)	TON^c^ (g/g)	Enzyme loading (mg/mL)	yield (%)	purity (%)	titer (mM)	E‐factor^e^	Auxiliary reagents	Reference
2	YjjG, UP, DeoB, YeiN	**UMP**	**Ψ**	18/74	22	9.0	80	80^d^	800	3.4^f^	Mn^2+^	Pfeiffer et al. (2023)
1	UP, DeoB, YeiN	**U**	**ΨMP**	30/93	74	4.3	97	95	970	2.4	Mn^2+^	Pfeiffer et al. (2023)
4	RbsK, AcK, YeiN	**Rib, Ura**	**ΨMP**	38/117	146	2.0	90	70^d^	650	43^f^	ATP, AcP, Mg^2+^	Ribar et al. (2024)
5	RY, YeiN	**UMP**	**ΨMP**	294/906	103	3.0	95	95	950	3.0	Mn^2+^	This study

^a^ Route identifiers as depicted in Figure 1.

^b^ Enzyme names used: RY, R341A‐Y347A double variant of PpnN (EC 3.2.2.10); UP, **U** phosphorylase (EC 2.4.2.3); DeoB, phosphopentomutase (EC 5.4.2.7); YjjG, nucleotide 5′‐phosphate phosphatase (EC 3.1.3.5); RbsK, ribokinase (EC 2.7.1.15); AcK, acetate kinase (EC 2.7.2.1); YeiN, **ΨMP**‐*C*‐glycosidase (EC 4.2.1.70).

^c^ The mass‐based TON (turnover number) is the gram amount of product produced and enzyme used.

^d^ Compound was not isolated, purity refers to its relative abundance in the reaction mixture.

^e^ The E‐factor is the ratio of the mass of waste per mass of product. The enzyme production is excluded from the calculations. See the Supporting Information (E‐factor calculation file).

^f^ The E‐factor was calculated under exclusion of the product isolation. See the Supporting Information (E‐factor calculation file).

## 4 Conclusions

A new cascade transformation of **UMP** to obtain **ΨMP** in a perfectly atom‐economic N‐C β‐riboside rearrangement was presented. The transformation was realized by coupling the nucleosidase reaction of PpnN and the C‐C condensation reaction of YeiN. The R341A‐Y347A double variant of *E. coli* PpnN was less inhibited by **Rib5P** than the wild‐type enzyme and was therefore preferred for usage together with YeiN in one‐pot conversions of **UMP** into **ΨMP**. As a strategy to minimize inhibition caused by accumulation of the intermediary **Rib5P**, reactions were performed using YeiN in excess over PpnN. **ΨMP** was obtained in ≥ 95% yield at over 300 g/L product with superior productivity of 2.9 × 10^2 ^g/L/h. The product received after enzyme removal and lyophilization showed high technical‐grade purity (≥ 93%). Gram amounts of **ΨMP** were obtained from cascade reaction in only 5.0 mL of total volume. Overall, the results of this study emphasize the huge potential of biocatalysis to enable development of more efficient and eco‐friendly manufacturing processes for pharmaceutical production (Benítez‐Mateos et al. 2022; France et al. 2023; Pollard and Woodley 2007).

## Author Contributions

**Martin Pfeiffer** and **Bernd Nidetzky:** design of study. **Franziska Guld:** experiments. **Franziska Guld** and **Martin Pfeiffer:** data analysis. **Martin Pfeiffer** and **Bernd Nidetzky:** supervision. **Martin Pfeiffer** and **Bernd Nidetzky:** manuscript writing. **Bernd Nidetzky:** funding acquisition.

## Conflicts of Interest

The authors declare no conflicts of interest.

## Supporting information

E‐factor calculation.

SI Revison May13.

## Data Availability

The data that support the findings of this study are available from the corresponding author upon reasonable request.
