# Supplementary material for: N‐ to C‐Glycoside Rearrangement of Uridine 5′‐Phosphate in Two Enzymatic Steps for the Production of Pseudouridine 5′‐Phosphate
Source: Biotechnol Bioeng. 2025 May 28;122(9):2456–64. doi: 10.1002/bit.29037 (PMC12322656; doi:10.1002/bit.29037)
Supplement: Supplementary file 2 — SI Revison May13. [file BIT-122-2456-s002.docx]

**Supporting Information**

***N*- to *C*-glycoside rearrangement of uridine 5’-phosphate in two enzymatic steps for the production of pseudouridine 5’-phosphate**

Martin Pfeiffer,^1^ Franziska Guld,^1^ Bernd Nidetzky^1,2,*^

^1^Institute of Biotechnology and Biochemical Engineering, Graz University of Technology, NAWI Graz, Petersgasse 12, A-8010 Graz, Austria

^2^Austrian Centre of Industrial Biotechnology (acib), Krenngasse 37, A-8010 Graz, Austria

* Corresponding author (B.N.; email: bernd.nidetzky@tugraz.at)

## Table of Content

[1.Supporting Methods 3](#_Toc194700480)

[1.1 Cloning and site-directed mutagenesis 3](#_Toc194700481)

[1.2 Enzyme expression and purification 4](#_Toc194700482)

[1.3 Nuclear magnetic resonance spectroscopy 5](#_Toc194700483)

[1.4 High performance liquid chromatography (HPLC) 6](#_Toc194700484)

[2. Supporting Figures 7](#_Toc194700485)

Figure S1. SDS polyacrylamide gel showing the purified enzymes used in this study 7

Figure S2. Dependence of the **UMP** hydrolysis activity of wild-type PpnN and RY on the pH 8

Figure S3. Dependence of the **UMP** hydrolysis activity of wild-type PpnN and RY on the temperature 9

Figure S4. Michaelis Menten plot of wild-type PpnN (a) and RY (b). 10

Figure S5. Analysis of product inhibition by **Rib5P** for wild-type PpnN (a) and RY (b) 11

Figure S6. Modified Dixon plot for the visualization of the inhibition by **Rib5P** in wild-type PpnN (a) and RY (b) 12

Figure S7. Effect of the mass ratio of enzymes used in the cascade reaction on the productivity of **ΨMP** release 13

Figure S8. Intensification of the RY-YeiN cascade reaction for **ΨMP** production 14

Figure S9. ^1^H NMR data for the synthesized **ΨMP** 15

Figure S10. ^13^C NMR data for the synthesized **ΨMP** 16

## 1.Supporting Methods

### 1.1 Cloning and site-directed mutagenesis

The gene of PpnN was ordered from Genescript (Leiden, The Netherlands) with overhangs at the 5`- (5’-CTAGAAATAATTTTGTTTAACTTTAAGAAGGAGATATA

CC-3’) and 3`- (5’-CTAGAAATAATTTTGTTTAACTTTAAGAAGGAGATATACC-3’) ends complementary to a linearized pET28a(+) vector. Linearization of pET28a(+) was achieved by PCR using the primer pair pET28a(+)_fwd/rev (**Table S1**) and Q5-polymerase (NEB, Ipswich, MA, USA) following the manufacturer’s instructions and a temperature profile consisting of 98°C/15 s initial denaturation; 25 cycles of 98°C/5 s denaturation, 65°C/15 s annealing, 72°C/180 s elongation; and a final extension step 72°C/1 min. The PCR product was digested with DpnI and purified using NEB PCR cleanup kit. The gene fragment and the linear vector were assembled using Gibson NEB builder^®^ (NEB, Ipswich, MA, USA) and transformed into *E. coli* NEB 10-beta and selected on LB agar plates with 0.05 mg/mL kanamycin. From single colonies overnight cultures (ONC) were prepared in LB medium with 0.05 mg/mL kanamycin, and plasmid was isolated using the Wizard® Plus SV Minipreps (Promega, Madison, WI, USA).

The double variant R341A_Y347A was created by site-directed mutagenesis using the Q5 Site-Directed Mutagenesis Kit^®^. The pET28a(+) plasmid harboring the PpnN gene was amplified by PCR using the mutagenesis primers (see **Table S1**) and Q5-polymerase (NEB), using a temperature profile consisting of 98°C/15 s initial denaturation; 15 cycles of 98°C/5 s denaturation, 55°C/15 s annealing, 72°C/180 s elongation; and a final extension step 72°C/180 s.

The PCR product was treated with the KLD-enzyme mix according to the manufacturer’s instruction and 1.0 µL of the reaction was transformed into *E. coli* NEB 10-beta and selected on LB agar plates with 0.05 mg/mL kanamycin. From single colonies, overnight cultures (ONC) were prepared in LB medium with 0.05 mg/mL kanamycin, and the plasmid was isolated.

Incorporation of the mutation was confirmed by sequencing (Balgach, Switzerland) and the vector was transformed into *E. coli* NiCo21 (DE3) cells and selected on LB agar plates with 0.05 mg/mL kanamycin. From a single colony a glycerol stock was prepared, and stored at -80°C until further use.

Table S1 Primers used in this study. Nucleotide triplets introducing mutations are underlined.

| **Primer name** | **Sequence (5’ to 3’)** |
| --- | --- |
| pET28a(+)_fwd | CACCACCACCACCACCAC |
| pET28a(+)_rev | CATGGTATATCTCCTTCTTAAAGTTAAACAAAATTATTTCTAG |
| R341A1_fwd | agaaaatcgcgcggatacaggcgatgc |
| R341A1_rev | ttcaccagcggcatc |
| Y347A2_fwd | aggcgatgccgcgagctttaactgg |
| Y347A2_rev | gtatcacggcgattttc |

### 1.2 Enzyme expression and purification

Enzymes were expressed in 1-L baffled shake flasks at 37 °C and 110 rpm, using 250 mL LB medium with 0.05 mg/mL kanamycin. Cultures were started from overnight cultures that were inoculated from glycerol stocks to an OD600 of 0.1 and grown until reaching an OD600 of ~0.8. At this point the temperature was reduced to 18 °C, and expression was induced with 0.4 mM IPTG for 20 h. Cells were harvested by centrifugation at 4420 × g for 30 min at 4 °C using a Sorvall RC-5B refrigerated superspeed centrifuge (Du Pont Instruments, Newtown, CT, USA), and the supernatant was discarded.

Cell pellets containing His_6_-tagged proteins were resuspended in His-tag binding buffer (50 mM HEPES, pH 8.0, containing 500 mM NaCl, 30 mM imidazole, 5% glycerol). Cells were disrupted by sonication (Fisherbrand Sonic Dismembrator, Ultrasonic Processor FB-505; Fisher Scientific, Vienna, Austria) for 6 min on ice, and the cell-free supernatant was collected by centrifugation at 27,150 × g for 50 min at 4 °C.

Enzymes were purified using immobilized metal affinity chromatography. The cell lysate (20 mL) was loaded onto two 5 mL HisTrap FF columns (Cytiva, Marlborough, MA, USA) equilibrated with His-tag binding buffer and mounted on an ÄKTA prime plus system (Cytiva). Purification was conducted at 10 °C with a flow rate of 3.0 mL/min. Protein elution was achieved using an imidazole gradient from 0% to 100% with His-tag elution buffer (50 mM HEPES, pH 8.0, 500 mM NaCl, 300 mM imidazole). Fractions containing the target protein were pooled, concentrated, and buffer-exchanged using Amicon Ultra-15 Centrifugal Filter Units (Millipore, Billerica, MA, USA). The final protein concentration was adjusted to 20–50 mg/mL in 50 mM HEPES buffer (pH 7.0) containing 5% glycerol (by volume), 200 mM NaCl. The enzyme was stored at -20 °C until further use.

### 1.3 Nuclear magnetic resonance spectroscopy

To remove Mn^2+^, the NMR sample (600 µL, 200 mM **ΨMP**) was treated with 50 mg Amberlite IRC120H and neutralized with NaOH before free drying. The resulting white solid was resolubilized in D_2_O. NMR spectra were recorded on a JEOL JNM-ECZL 400 MHz NMR Spectrometer (^1^H: 399.78 MHz, ^13^C: 100.53 MHz, ^31^P: 161.83 MHz).

Chemical shifts δ are referenced to the residual proton and carbon signal of the deuterated solvent D_2_O: δ = 4.79 ppm (^1^H). Chemical shifts δ are given in ppm (parts per million) and coupling constants *J* in Hz (Hertz). Signal multiplicities are abbreviated as s (singlet), d (doublet), t (triplet), m (multiplet), dd (doublet of doublets).

### 1.4 High performance liquid chromatography (HPLC)

Samples from enzymatic reactions were analyzed by reversed-phase ion-pairing HPLC. Ten μL of sample containing ~1.0 mM of the analyte was loaded on a Kinetex C18 EVO column (Phenomenex, Aschaffenburg, Germany; 5 μm, 100 Å, 150 × 4.6 mm). Analytes were separated in 15-min long isocratic runs using 20 mM phosphate buffer, pH 5.9, containing 40 mM tetra-n-butylammonium bromide. The flow rate was 0.25 mL/min and the temperature was 35 °C. Detection was at 260 nm. Retention times were as follows: **Ψ** (7.1 min), **Ura** (7.4 min) and **ΨMP** (13.5 min).

## 2. Supporting Figures


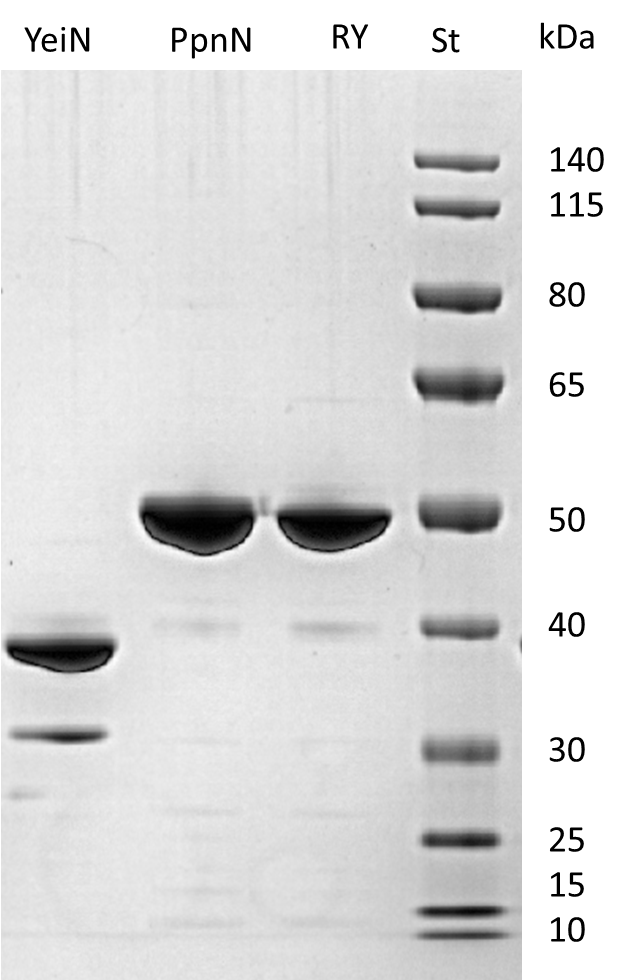


Figure S1. SDS polyacrylamide gel showing the purified enzymes used in this study. ΨMP-C-glycosidase (YeiN); wild-type purine/pyrimidine nucleoside 5’-phosphate hydrolase (PpnN); R341A-Y347A double variant of PpnN (RY); St, standard, Pre-stained Page Ruler (Thermo-Scientific, Waltham, MS, USA).





Figure S2. Dependence of the UMP hydrolysis activity of wild-type PpnN and RY on the pH. Relative activity (%) was normalized to the activity measured at pH 7.5 for each enzyme (wild-type PpnN: 2.6 ± 0.8 U/mg; RY: 9.6 ± 1.6 U/mg). Reactions contained 50 mM HEPES and 30 mM UMP. Wild-type PpnN was used at 0.40 mg/mL. RY was used at 0.10 mg/mL. Reactions were performed in 100 µL and incubated at 40°C. Error bars show the standard deviation from at least three replicate experiments.





Figure S3. Dependence of the UMP hydrolysis activity of wild-type PpnN and RY on temperature. Relative activity (%) was normalized to the activity measured at 40 °C for each enzyme (wild-type PpnN: 2.6 ± 0.8 U/mg; RY: 9.6 ± 1.6 U/mg). Reactions were performed in 50 mM HEPES buffer, pH 7.5, and contained 30 mM UMP. Wild-type PpnN was used at 0.30 mg/mL. RY was used at 0.10 mg/mL. Error bars show the standard deviation from at least three replicate experiments.


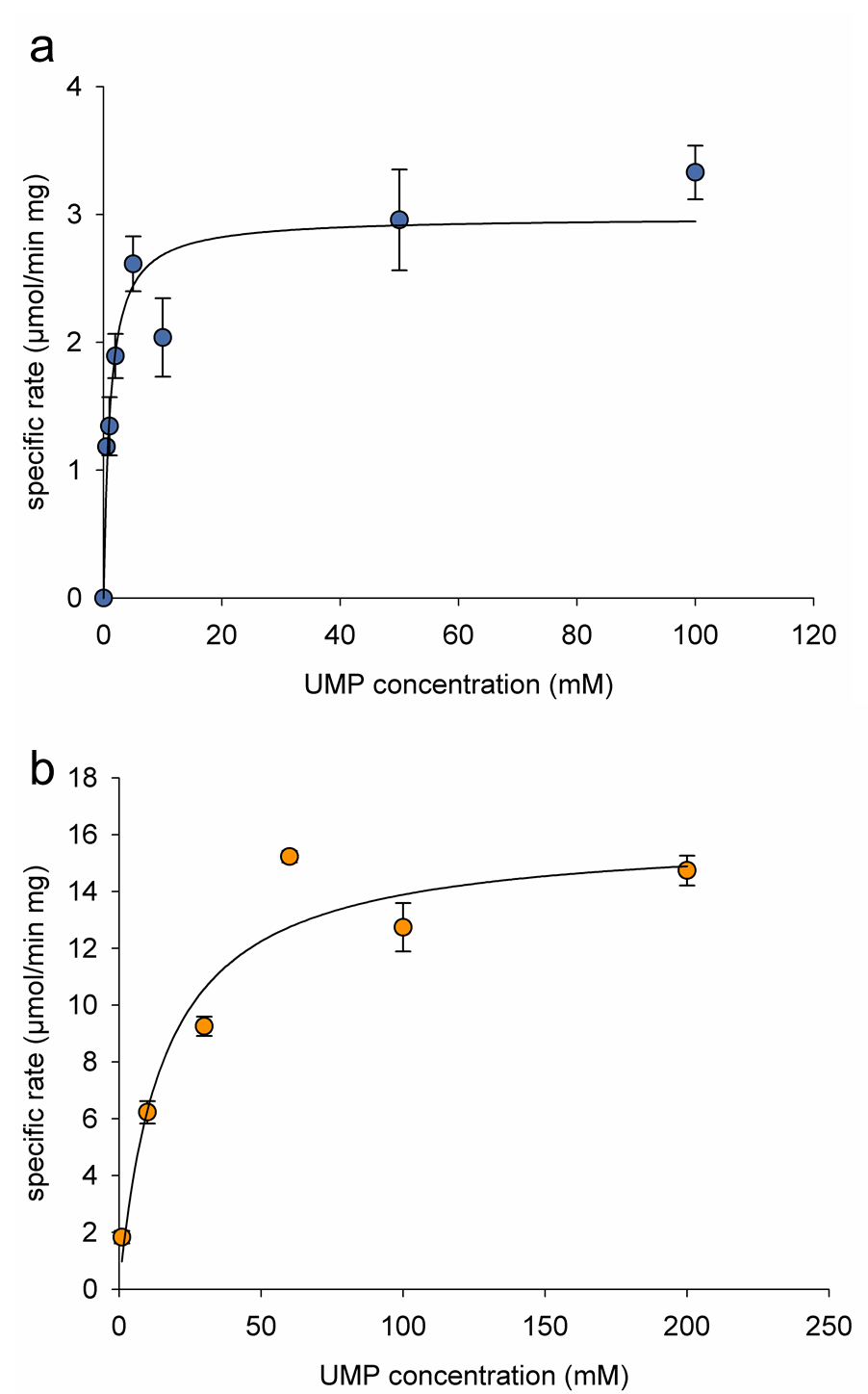


Figure S4. Michaelis-Menten plot for wild-type PpnN (a) and RY (b). Reactions were performed in 50 mM HEPES, pH 7.5, at 40°C. The UMP concentration was varied in the range of 0.5 - 100 mM for wild-type PpnN and 1.0 - 200 mM for RY. The enzyme concentration was adjusted to allow for convenient measurement of the initial rate at the different substrate concentrations, i.e., 0.03 - 0.60 mg/mL for wild-type PpnN and 0.03 - 0.30 mg/mL for RY. The initial rates shown in the graphs were normalized to an enzyme concentration of 1.0 mg/mL and are termed specific rates. Error bars show the standard deviation from three replicate experiments.


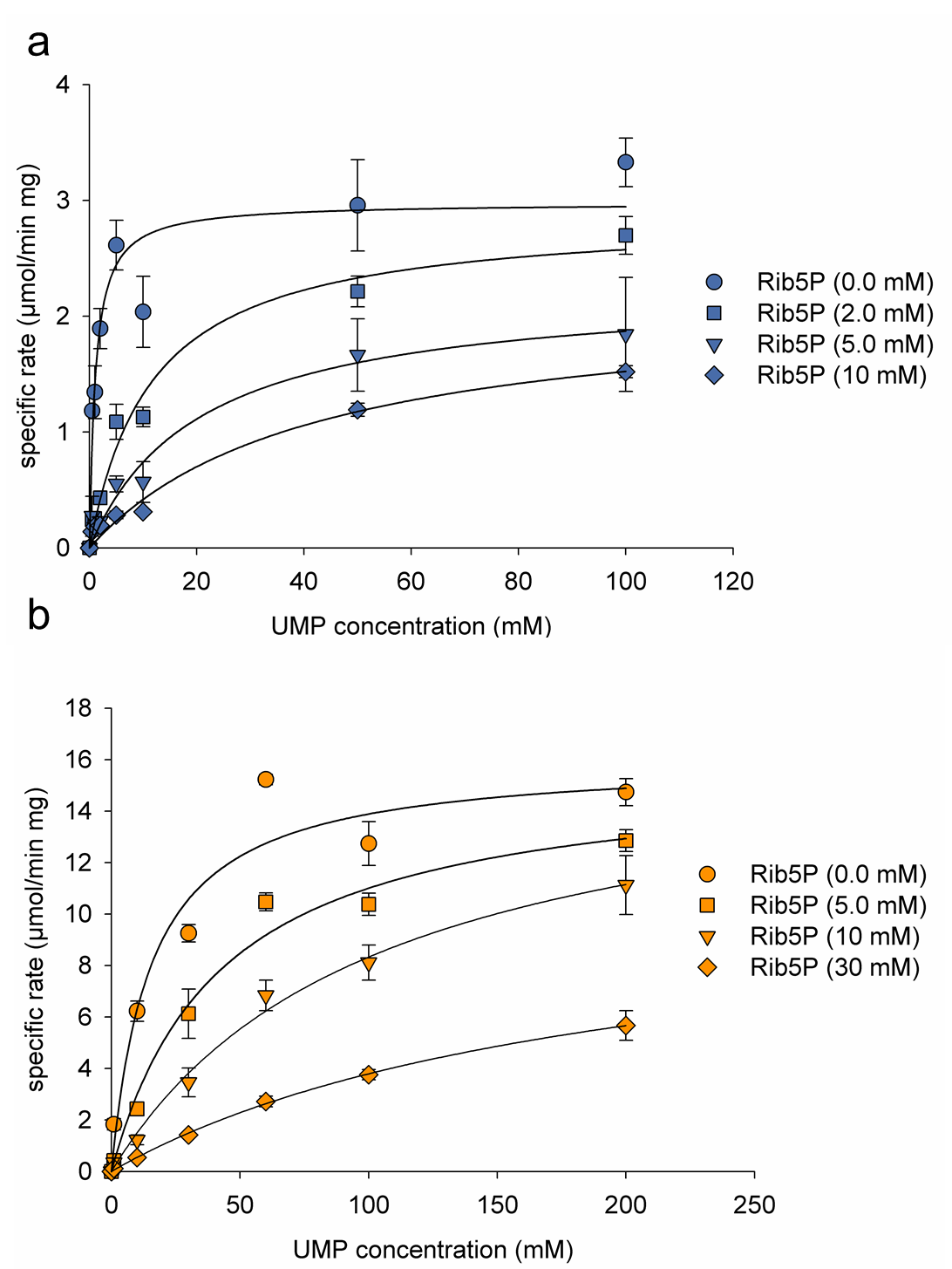


Figure S5. Analysis of product inhibition by Rib5P for wild-type PpnN (a) and RY (b). Reactions were performed at 40°C in 50 mM HEPES buffer, pH 7.5. The concentrations of UMP and Rib5P were varied as indicated in the graphs. For UMP, the lowest concentration was 0.5 mM for wild-type PpnN and 1.0 mM for RY. The enzyme concentration was adjusted to allow for convenient measurement of the initial rate at the different substrate concentrations, i.e., 0.03 - 0.60 mg/mL for wild-type PpnN and 0.03 - 0.30 mg/mL for RY. The initial rates shown in the graphs were normalized to an enzyme concentration of 1.0 mg/mL and are termed specific rates. Error bars show the standard deviation from three replicate experiments.


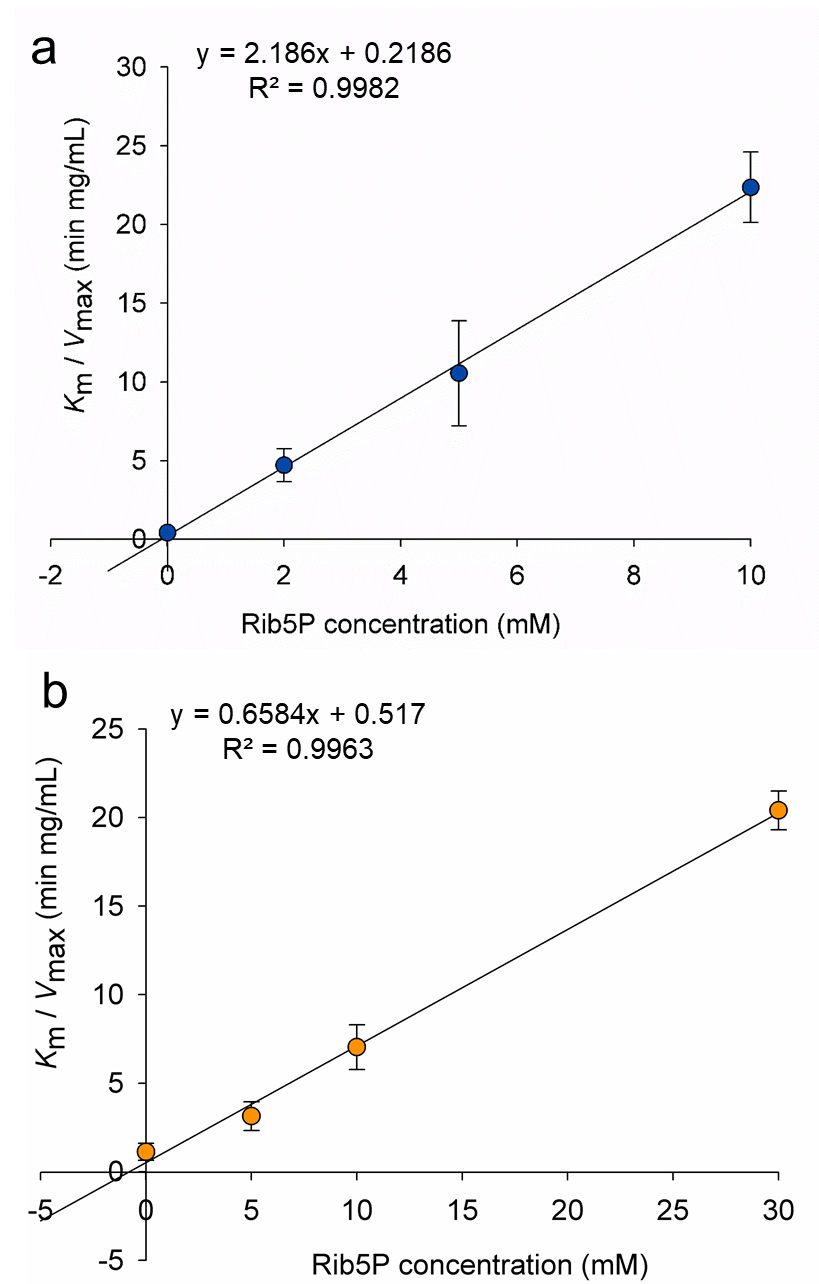


Figure S6. Modified Dixon plot for the visualization of the inhibition by Rib5P in wild-type PpnN (a) and RY (b). The intercept with the abscissa gives -K_i_. The V_max_ and K_m_ were derived from fits of the individual Michaelis-Menten plots shown in Figure S5. Error bars show the standard deviation from three replicate experiments.


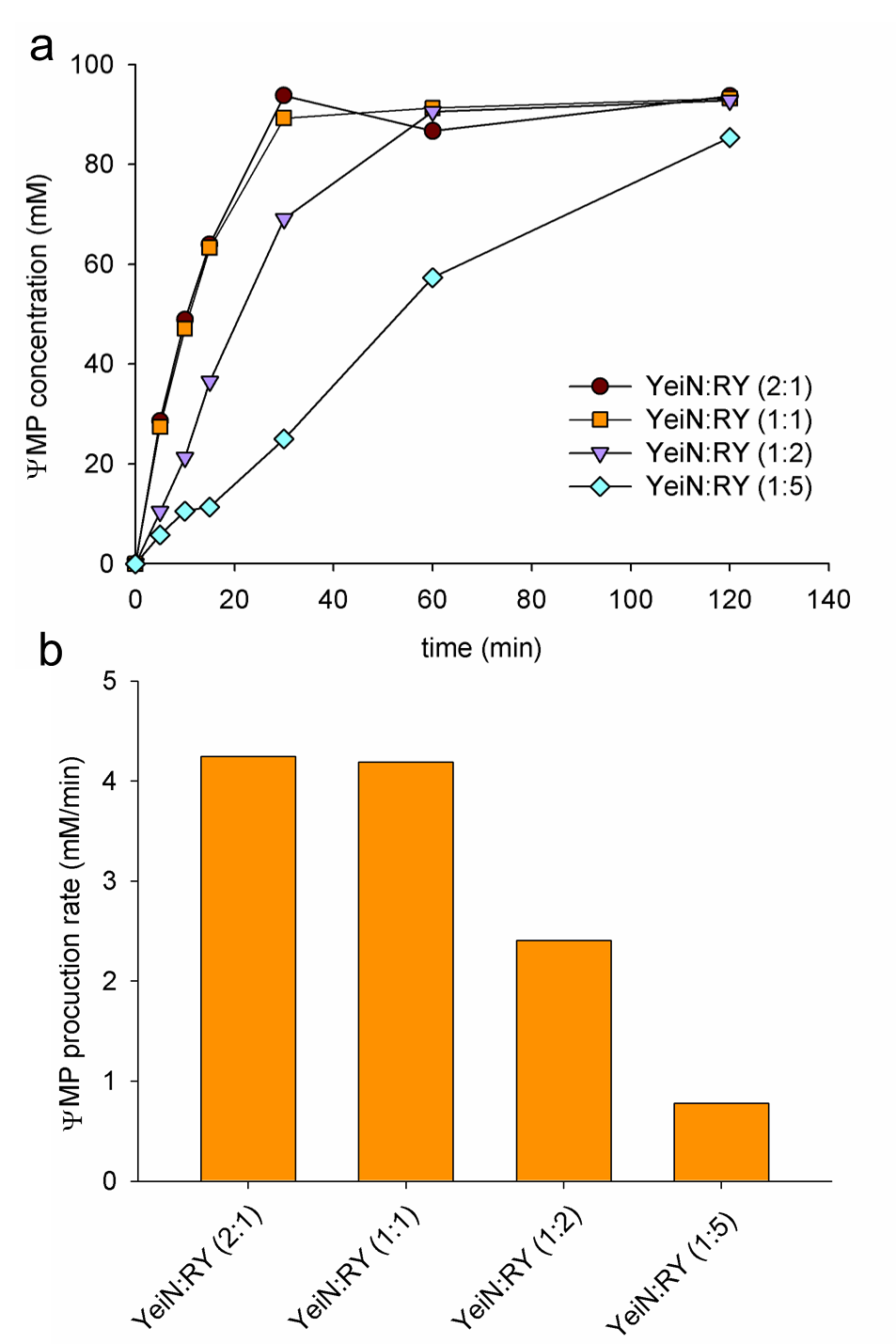


Figure S7. Effect of the mass ratio of enzymes used in the cascade reaction on the productivity of ΨMP release. a) Time courses of the ΨMP formation and b) analysis of the production rate. The reactions were performed at 40°C in a total volume of 300 µL. A 50 mM HEPES buffer (pH 7.5) was used with 100 mM UMP and 15 mM MnCl₂. RY was used at 0.50 mg/mL RY whereas YeiN was varied at 1.00, 0.50, 0.25 or 0.10 mg/mL.


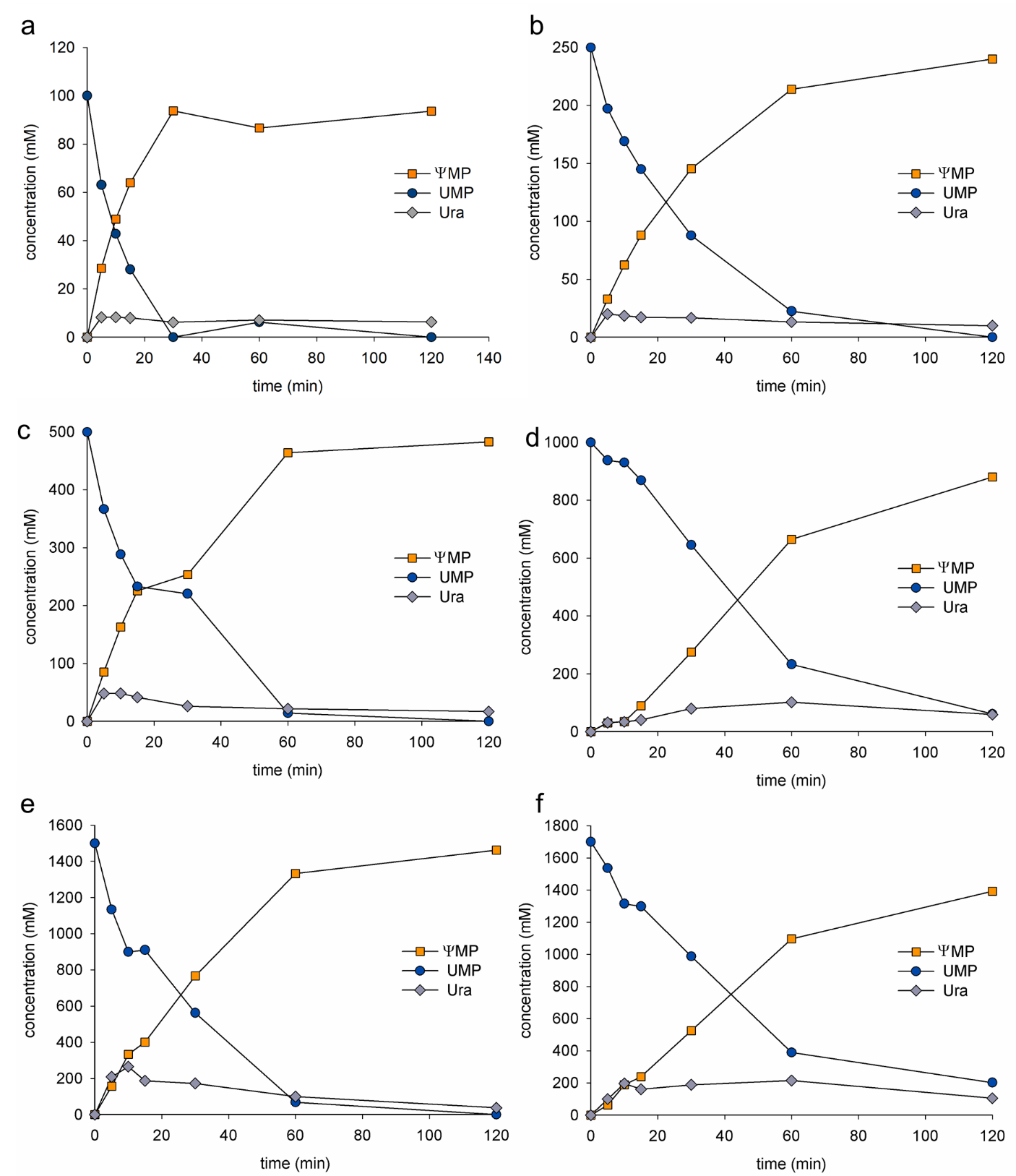


Figure S8. Intensification of the RY-YeiN cascade reaction for ΨMP production. The reactions were performed at 40°C in a total volume of 300 µL. A 50 mM HEPES (pH 7.5) was used with 15 mM MnCl₂. The UMP concentration was varied between 0.1 and 1.7 M (a-f). The enzyme concentrations were adjusted based on the UMP concentration used: 0.1 - 0.2 M UMP, 0.50 mg/mL RY, 1.0 mg/mL YeiN; 0.5 - 1.0 M UMP, 1.0 mg/mL RY, 2.0 mg/mL YeiN; >1.0 M UMP, 2.0 mg/mL RY, 4.0 mg/mL YeiN.


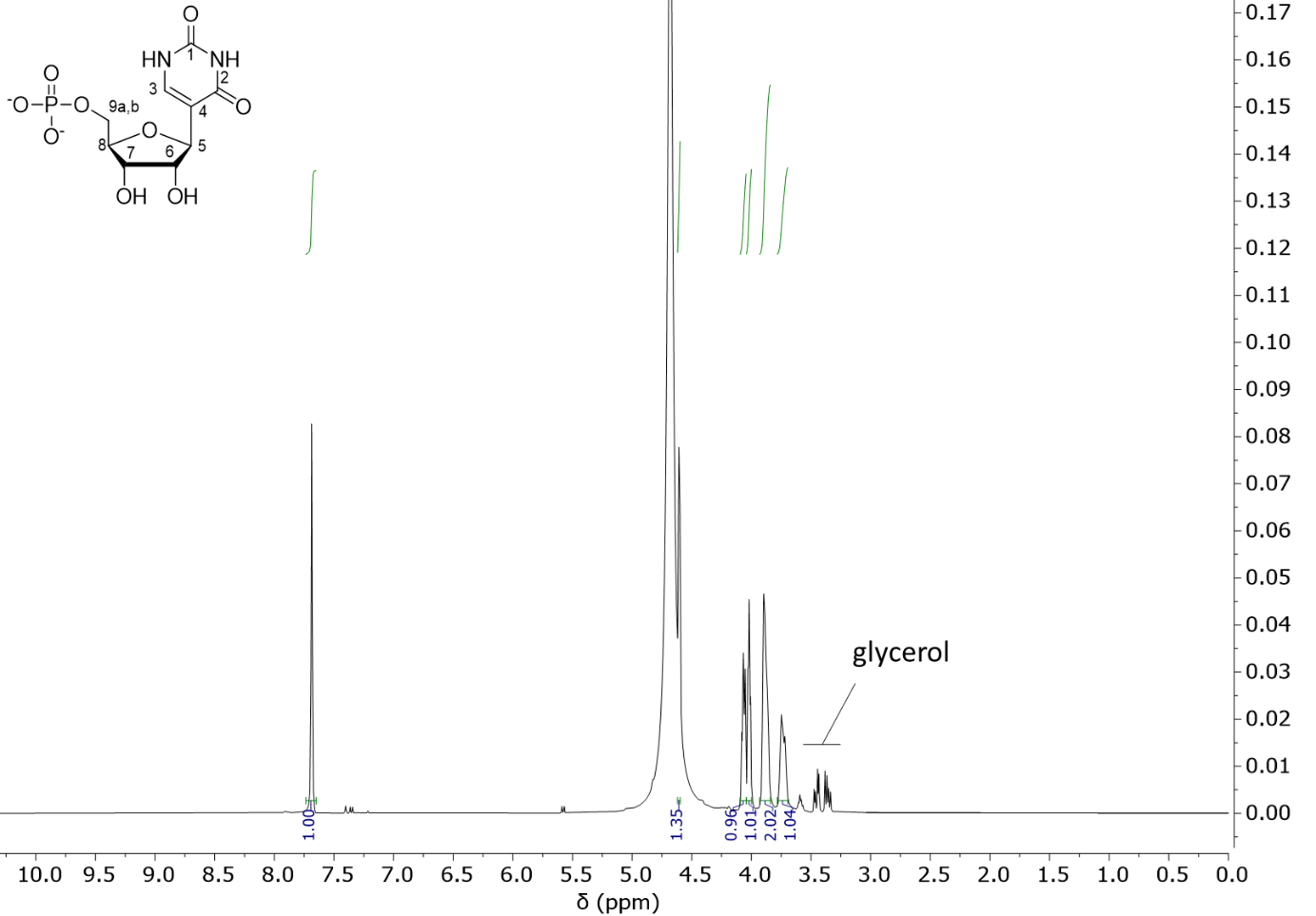


Figure S9. ^1^H NMR data for the synthesized ΨMP. ^1^H-NMR (400 MHz, D_2_O). δ = 7.69 (s, 1H, H-3), 4.60 (d, J = 3.4 Hz, H-5), 4.07 (t, J = 5.9 Hz, 1H, H-7), 4.02 (d, J = 4.0 Hz, 1H, H-6), 3.93 – 3.84 (m, 2H, H-8, H-9), 3.73 (dd, J = 11.6, 5.0 Hz, 1H, H-9). Glycerol (10 mol %).


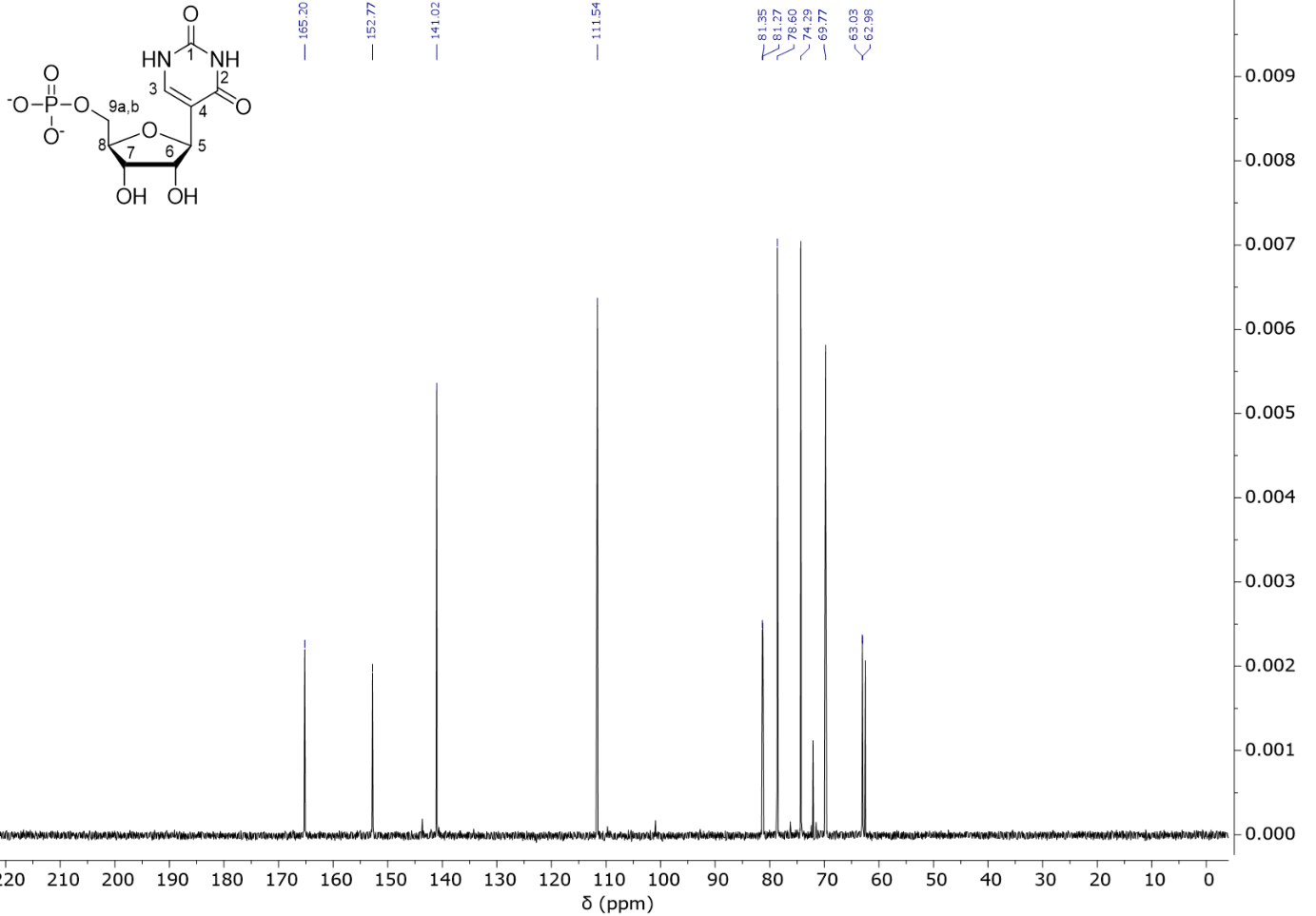


Figure S10. ^13^C NMR data for the synthesized ΨMP. ^13^C-NMR (101 MHz, D_2_O). δ = 165.20 (C_q_, C-1), 152.77 (C_q_, C-2), 141.02 (CH, C-3), 111.54 (C_q_, C-4), 81.31 (d, J = 8.3 Hz CH, C-8), 78.60 (CH, C-5), 74.29 (CH, C-6), 69.77 (CH, C-7), 63.00 (d, J = 4.4 Hz, CH_2_, C-9).
